# The Antioxidative Effects of Picein and Its Neuroprotective Potential: A Review of the Literature

**DOI:** 10.3390/molecules27196189

**Published:** 2022-09-21

**Authors:** Leila Elyasi, Jessica M. Rosenholm, Fatemeh Jesmi, Mehrdad Jahanshahi

**Affiliations:** 1 Neuroscience Research Center, Department of Anatomy, Faculty of Medicine, Golestan University of Medical Sciences, Gorgan 4917955315, Iran; 2 Pharmaceutical Sciences Laboratory, Faculty of Science and Engineering, Åbo Akademi University, 20500 Turku, Finland; 3 Pars Advanced and Minimally Invasive Medical Manners Research Center, Pars Hospital, Iran University of Medical Sciences, Tehran 1415944911, Iran

**Keywords:** neurodegenerative diseases, neuroprotective agents, picein

## Abstract

Neurodegenerative diseases (NDDs) are the main cause of dementia in the elderly, having no cure to date, as the currently available therapies focus on symptom remission. Most NDDs will progress despite treatment and eventually result in the death of the patient after several years of a burden on both the patient and the caregivers. Therefore, it is necessary to investigate agents that tackle the disease pathogenesis and can efficiently slow down or halt disease progression, with the hope of curing the patients and preventing further burden and mortality. Accordingly, recent research has focused on disease-modifying treatments with neuroregenerative or neuroprotective effects. For this purpose, it is necessary to understand the pathogenesis of NDDs. It has been shown that oxidative stress plays an important role in the damage to the central nervous system and the progression of neurodegenerative disorders. Furthermore, mitochondrial dysfunction and the accumulation of unfolded proteins, including beta-amyloid (Aβ), tau proteins, and α-synuclein, have been suggested. Accordingly, cellular and molecular studies have investigated the efficacy of several natural compounds (herbs and nutritional agents) for their neuroprotective and antioxidative properties. The most popular herbs suggested for the treatment and/or prevention of NDDs include *Withania somnifera* (ashwagandha), ginseng, curcumin, resveratrol, *Baccopa monnieri*, and *Ginkgo* *biloba*. In some herbs, such as ginseng, preclinical and clinical evidence are available for supporting its effectiveness; however, in some others, only cellular and animal studies are available. In line with the scant literature in terms of the effectiveness of herbal compounds on NDDs, there are also other herbal agents that have been disregarded. Picein is one of the herbal agents that has been investigated in only a few studies. Picein is the active ingredient of several herbs and can be thus extracted from different types of herbs, which makes it more available. It has shown to have anti-inflammatory properties in cellular and plant studies; however, to date, only one study has suggested its neuroprotective properties. Furthermore, some cellular studies have shown no anti-inflammatory effect of picein. Therefore, a review of the available literature is required to summarize the results of studies on picein. To date, no review study seems to have addressed this issue. Thus, in the present study, we gather the available information about the antioxidative and potential neuroprotective properties of picein and its possible effectiveness in treating NDDs. We also summarize the plants from which picein can be extracted in order to guide researchers for future investigations.

## 1. Background and Motivation

Neurodegenerative diseases (NDDs) are one of the main concerns of modern medicine and are responsible for the majority of the 50 million cases of dementia worldwide [1]. NDDs are progressive diseases that result in a great disease burden and as most NDDs are age-related diseases [2], their incidence and disease burden are expected to increase annually due to an aging population and triple by 2050 [3]. Alzheimer’s disease (AD) is the most commonly known NDD, although there are also other types of NDDs. These include, but are not limited to Parkinson’s disease (PD), multiple sclerosis (MS), amyotrophic lateral sclerosis (ALS), and Huntington’s disease [4]. Despite the different clinical, laboratory, and neuroimaging features, as well as the neuropathology and management of NDDs, they all have the common feature of neurodegeneration (gradual loss of specific neuronal populations), which makes them progressive diseases that result in the death of patients after several years of suffering (for the patient as well as their caregivers). Despite advances in medicine, there is still no cure for NDDs and available medications can only relieve the patient’s symptoms and improve brain function deficits (memory, movement, and cognition). Sometimes the treatments are inefficient due to the multiplicity and complexity of the patient’s symptoms [5,6]. Therefore, disease-modifying treatments, which can efficiently slow down or halt disease progression, have been identified as the main treatment goal in NDDs, for which several trials have been performed, although the progression rate seems slow [7,8]. Most recent research has focused on neuroprotective agents to target the pathophysiology of NDDs [9].

Considering the unmet need for effective curative treatment of NDDs, in this paper, we provide a short review of the bioactive compounds and herbal agents with potentially neuroprotective properties that have been examined in preclinical and clinical studies of NDDs with the main focus on an agent that has been scarcely evaluated, picein. As far as we are concerned, this is the first review of the potential neuroprotective effects of this herbal agent. In the final section, we outline the benefits of picein on plants because of the scant evidence available in animal and human studies and the favorable outcomes reported in plant studies. To initiate this review, we provide an overview of the pathogenesis of NDDs in order to understand how this treatment agent can influence the disease pathologies.

## 2. Pathogenesis of Neurodegenerative Diseases

One of the main pathologies of NDDs is related to oxidative stress induced by the excessive amount of oxygen byproducts and the nitrogen-rich tissue environment, including nitric oxide (NO), reactive nitrogen species (RNS), and reactive oxygen species (ROS), which result in high oxygen consumption, low antioxidant defenses, and high content of polyunsaturated fats in the central nervous system (CNS) [10]. It should be noted that free radicals act as a double-edged sword and their excess, in addition to their elimination, causes problems for humans; low levels of free radicals are necessary for several functions in the body, such as the regulation of critical signaling pathways involved in cognitive function, cell growth, proliferation, differentiation and survival, regulation of blood pressure, and immunity [11]. Furthermore, RNS have additional roles in the CNS including regulating cerebral blood flow and memory and maintaining the immune system and cytokine production [12]. On the other hand, excess ROS and RNS, if not neutralized by endogenous antioxidants, result in oxidative stress [13]. In physiological conditions, the normal antioxidant defense mechanisms of the body are maintained through the balance between the enzymatic and non-enzymatic antioxidant systems and ROS through which oxidative stress is prevented [14]. However, exposure to oxidative damage due to age and/or environmental factors, such as irradiation, causes an accumulation of RNS and ROS, which can alter the cell membrane permeability, cause mitochondria injury and DNA mutation, impair the mitochondrial respiratory chain, change the membrane permeability influencing Ca^2+^ homeostasis, increase intracellular concentrations of free calcium, release excitatory amino acids ending in autophagy and apoptosis, cause irreversible damage to the glial cells and neurons, and induce neurodegeneration [15]. Humans can be exposed to free radicals through several mechanisms, such as aging and inflammation, and environmental factors, such as nutrition, stress, and viruses, which result in further ROS accumulation and make NDDs a progressively deteriorating disease [16]. Considering mitochondrial dysfunction as a leading cause of oxidative stress, novel antioxidative treatments have shown favorable outcomes in the alleviation of oxidative stress and the return of balance, resulting in attenuated mitochondrial dysfunction and cell degeneration [17]. In an oxidative stress-free environment, the differentiation of macrophages is promoted, preferentially to the M2 phenotype, which contains growth factors and can even promote healing [18]. Accordingly, preclinical and clinical studies have examined the effects of several natural and synthetic antioxidants on the treatment of NDDs [19,20]. 

In addition to the direct neuronal damage induced by oxidative stress, other important mechanisms responsible for the pathological changes during NDDs with a causative role in most NDD symptoms, are the disturbance of metal metabolism and aggregation of misfolded proteins. These misfolded proteins are physiologically present in neurons and play several physiological roles in the CNS and healthy subjects, cells have a quality control system for the maintenance of homeostasis that acts as a defense mechanism against aggregated misfolded proteins (into a β-sheet structure). The chaperons are responsible for refolding, degrading, or sequestering the misfolded proteins for the elimination of aggregated forms of abnormal, pathogenic, and toxic proteins. Nonetheless, in pathologic conditions, the aggregation of their misfolded form results in cytotoxicity and cell death. Most of these proteins can form amyloid fibrils, which have amino acid sequences that will aggregate into cytotoxic amyloid fibrils in pathologic conditions [21]. The amyloid aggregates are extensively stable thermodynamically and resist further degradation. They can also convert native proteins to amyloid forms, which results in amyloid fibrils and plaques. If these defense systems are impaired by oxidative stress or any other mechanism, it can ultimately result in an NDD [22]. Evidence shows that an ROS imbalance results in a vicious cycle in the aggregation of misfolded proteins, as oxidative stress is responsible for the generation and aggregation of misfolded proteins, and the misfolded proteins increase ROS production and neurotoxicity [23].

These misfolded proteins include beta-amyloid (Aβ), tau proteins, α-synuclein, polyglutamate, mutant huntingtin protein (mHtt), and ubiquitinated proteins [24]. Two of the most important misfolded proteins involved in AD include tau and Aβ. Tau proteins are microtubule-associated proteins (MAP) that maintain the stability and integrity of microtubules in axons through the regulation of the microtubule structure and dynamics by binding to the microtubule surface. This protein is natively unfolded and has little tendency for aggregation owing to its paperclip-like structure. In pathologic conditions, this structure is broken and the neutralization of the inhibitory domains causes tau proteins to assemble into neurofibrillary tangles (NFTs) that ultimately results in tau-induced toxicity [25]. The exact mechanism of tau accumulation is unclear, although the two suggested hypotheses include cleavage by endoproteolytic enzymes that can result in the dysfunction of tau proteins and disturbance to the mechanism of defense against tau accumulation (E3 ligase CHIP, Hsp70 and 90, which mediate ubiquitination of tau). Through accumulation, tau proteins shift to the somatodendritic compartment, become hyperphosphorylated, and aggregate into neurofibrillary tangles, which results in synaptic dysfunction [26]. 

Another important misfolded protein in NDDs is Aβ, which is produced under stress conditions by neurons and other cell types, including astrocytes and other glia, and plays an important role in brain development, memory, and synaptic plasticity. It is generated after proteolytic processing and sequential cleavage of type 1 membrane amyloid precursor protein (APP). APP is first cleaved by Beta Secretase-1 (BACE-1) enzymes to produce C99, a membrane-bound C-terminal fragment. Then, a second enzyme, γ secretase, cuts C99 to produce Aβ, and a third enzyme, α secretase, cleaves APP at a site within Aβ to preclude its formation. As β (BACE-1 ) and γ secretases are essential for the generation of Aβ, their inhibition can be a prime therapeutic target for NDDs, acting by decreasing the concentration of Aβ [27]. Furthermore, mutation of APP can result in upregulated production of Aβ, which causes extracellular aggregates of neuritic plaques composed of the Aβ peptide that cannot be properly degraded through the proteasome. Before forming insoluble fibrils, the pathogenic proteins aggregate into soluble toxic oligomers that expose hydrophobic surfaces and disturb the phospholipid bilayer, which results in autophagy and neurodegeneration [28]. Much effort has been directed toward the elimination or inhibition of the degradation of these pathogenic proteins using small molecules that induce autophagy to remove the misfolded proteins [22,28]. 

Neuro-inflammation caused by aggregated proteins activates inflammasomes, which function as intracellular sensors of danger and induce an innate immune response after activation. As CNS cells and microglia are prominent in innate immune cells, the activation of inflammasomes results in the secretion of inflammatory cytokines, including interleukins, which induces pyroptosis, a lytic cell death mode that releases additional inflammatory mediators. Neuroinflammation is considered the key pathogenesis in most NDDs, such as AD and PD, during which the microglia’s activity for Aβ phagocytosis is impaired, resulting in neuronal damage and synaptic dysfunction [29]. It has been demonstrated that Aβ-accumulated microglia have high levels of interleukin-1β that surround Aβ plaques with higher expression of other inflammatory markers such as caspase and NLRP3. In addition, in PD, aggregated α-synuclein contains increased concentrations of interleukin-1β that triggers the activation of NLRP3 inflammasome in monocyte and microglial cells. Activated caspase-1 has been also observed in the brains of patients with HD. These inflammasomes have a central role in neuro-inflammatory responses that can be also used as future novel therapeutic targets for NDDs [30].

In addition to the above, a specific role has been identified for the mitochondria in NDDs. The mitochondria are multifunctional organelles that play a role in oxygen- and proton-pump-driven ATP production through the establishment of an electrochemical gradient by respiratory chain proteins that can also trigger cell aging and death in pathologic conditions. Mutations in the mitochondria can cause several hereditary neuropathies such as optic atrophy type 1, Charcot-Marie-Tooth disease, Leber’s hereditary optic neuropathy, and progressive external ophthalmoplegia. Moreover, evidence shows mitochondrial involvement in NDDs, for instance, mitochondrial DNA and proteins are mutated in neurons of the substantia nigra of patients with PD. In addition, in ALS, mitochondrial respiratory chain enzymes are altered, which results in mitochondrial programmed cell death proteins. Furthermore, in AD, the mitochondrial binding of Aβ is altered [31]. The association of the mitochondria with NDDs has been related to the functional roles of the mitochondria in cell metabolic hemostasis, signaling, differentiation, and senescence, which helps maintain synaptic plasticity and neurotransmitter synthesis. The brain is one of the organs that requires a high energy source and is thus affected by mitochondrial dysfunction. Age has been found to be an independent driver for mitochondrial dysfunction, resulting in decreased membrane potential and reduced ATP synthesis. Moreover, the morphology of the mitochondria also changes with age and disorganizations accumulate in the mitochondria with age. Damage to mtDNA was also found to be correlated with the accumulation of oxidized proteins and increased oxidative stress and ROS production within the mitochondria through the switch from glycolysis to respiratory metabolism [10]. 

In the next section, we describe the mechanisms of some natural bioactive compounds, including herbs and nutritional agents, which have shown antioxidative properties and have been suggested as an effective preventive and treatment target for NDDs. 

## 3. Antioxidant and Neuroprotective Agents Suggested for the Treatment of Neurodegenerative Diseases

According to the progressive neuronal damage observed in NDDs and the significant effects of oxidative stress, the effectiveness of exogenous antioxidants able to neutralize free radicals and reduce plaque formation by affecting sequester metal ions are increasingly considered supplementary therapies for NDDs [32]. Several antioxidative agents, including nutritional and herbal medicines, have been investigated in cellular and molecular studies [19,20,33,34]. Several clinical trials have shown the efficacy of various medicinal plants on symptom relief and/or the prevention of neurological diseases. In a review of studies by Ratheesh et al., the most commonly used herbs were suggested to be Withania somnifera (ashwagandha), ginseng, curcumin, resveratrol, *Baccopa monnieri*, and *Ginkgo biloba* [35]. 

Methanol–chloroform and alcoholic extracts of Ashwagandha (the root extract of *Withania somnifera* Dunal) have shown anti-inflammatory, antioxidative stress, and neuroprotective properties, which activate choline acetyltransferase, inhibit the release of corticosterone, and boost serotonin in the hippocampus. The proposed mechanism may be effective in the downregulation of NO and neurochemical alterations in neurotransmitter systems and thus a potential treatment for NDDs [36,37]. Cellular and animal models have confirmed its efficacy on several NDDs. In cultured human neuroblastoma (NB) cells [38] and rat pheochromocytoma PC12 cell lines [39], Ashwagandha prevented Aβ-induced cytotoxicity. The upregulation of the peroxisome proliferator-activated receptor also supports the neuroprotective effect of this compound [38]. In addition, in a mouse model of parkinsonism, Withania somnifera root extract was able to decrease the level of catalase and lipid peroxidation, as well as tyrosine hydrolase in substantia nigra, in mice, which confirms the effectiveness of this agent against nigrostriatal dopaminergic neurodegeneration [40] and apoptotic pathways [41]. It was also shown to improve memory impairment in other brain diseases [42,43]. However, further studies are required to determine this compound as an effective treatment for NDDs in humans.

*Panax ginseng* is an ancient Korean and Chinese herbal medicine that has been used for thousands of years for the treatment of several diseases, including allergies, diabetes, hypertension, and cancer, with the ability to modulate neuronal calcium channels and immune modulation [44]. Not only animal studies [45] but also preclinical and clinical studies have confirmed the efficacy of the extract and powder and various ginsenosides of ginseng [46] on different NDDs, such as PD, AD, Huntington’s disease, ALS, and MS, through various mechanisms, including protection against neuronal toxicity, inhibition of caspase-3 and p-tau expression, and proinflammatory cytokines [47]. Improved AD scale, dementia, and mini-mental state scores have been documented after 12 weeks of using 9 gr/day Korean red ginseng powder or 4.5 gr/day Korean white ginseng powder [48,49]. In animals, the long-term use of ginseng total saponins (100 and 200 mg/kg/day) prevented memory loss [50]. From a cellular aspect, the neuroprotective effect of *Panax ginseng* has been related to the alleviation of 6-hydroxydopamine-induced neurotoxicity via the P13K/Akt/GSK-3 signaling pathway [51] and the regulation of the phosphate activity of calcineurin and tau protein [52]. Moreover, ginsenoside attenuates cognitive and memory impairment through increased levels of soluble amyloid precursor protein α, which exerts the anti-apoptotic and neuroprotective effects involved in the non-amyloidogenic pathway and reduction of Aβ [53]. As ginsenosides reveal antioxidative effects, as well as the modulation of intracellular neuronal signaling and metabolism, cell survival/death genes, and mitochondrial function, it has been suggested not only for the treatment but also for the prevention of NDDs [54].

Curcumin or turmeric (a dietary polyphenol and major constituent of *Curcuma longa* [Zingiberaceae]), indigenous to South and Southeast Asia, was and is still being used in India as a spice and includes fiber, potassium, magnesium, iron, and vitamins. It has been used in traditional medicine for several diseases, including cardiovascular, pulmonary, metabolic, autoimmune, and neoplastic diseases, with anti-inflammatory and antioxidative properties, a high potential for an immune boost, and downregulation of certain transcription factors, enzymes, and cytokines [55,56,57]. It also induces anti-inflammatory, anti-apoptotic, and neuroprotective properties through hyperphosphorylation and the clearing of Aβ plaques and tau protein by macrophages [58,59]. In vitro studies have shown that curcumin can inhibit the formation of Aβ fibrils in a dose-dependent manner; furthermore, some curcuminoid compounds (such as turmeric extract) suppressed Aβ aggregation and oligomerization. It has also been shown to be effective in reducing neuroinflammation (interleukins- 1α and 6 and TNF-α) [60,61]. Animal studies have also revealed the effect of curcumin on the nigrostriatal pathway in 6-hydroxydopamine-induced PD mediated by α7-nicotinic receptors, which modulate the immune system via the cholinergic anti-inflammatory pathway [62]. Clinical evidence has also proven the reduced frequency, severity, and duration of migraine attacks, lower scores on the revised ALS functional rating scale, and the occurrence of motor complications in PD with curcumin supplementation (alone or in combination with other ingredients) [63].

*Ginkgo biloba* is a popular dietary supplement that is used by the elderly for improving memory and cognitive dysfunction [64]. It has been available for over 200 million years and is indigenous to Korea, Japan, and China; however, Ginkgo is now found worldwide due to its favorable clinical effects that have been discovered in the last couple of decades. In addition to its effect on cerebral circulation, inhibition of platelet aggregation, and clot formation, it is also one of the most commonly used herbal remedies for AD and other NDDs with a positive effect on neuronal cell metabolism due to its antioxidative and neuroprotective properties [64,65,66]. The extract EGb 761 (Tanakan) is standardized to contain 24% flavonoids and 6% terpene lactones (3.1% ginkgolides and 2.9% bilobalide) and has been used in several investigations studying its effects [67]. The antioxidative effects of EGB 761 have been confirmed in several studies, which are attributed to the free radical scavenging properties of this compound and its protection against ROS and inflammasomes in the CNS [67]. Besides its antioxidative effect, it has been shown that Gingko can inhibit the production and aggregation of Aβ in the brain and inhibit its toxicity. The mechanism for restoring normal memory in rats has been suggested to be related to reducing the levels of free cholesterol, APP processing, and amyloidogenesis agent [64]. Several in vitro and in vivo studies have described the effect of Gingko against neuronal damage and cell death. The production of apolipoprotein E has been suggested as one of the mechanisms of neuroprotection for this substance [68,69]. Clinical trials have also proven its efficacy in several NDDs such as AD and PD [70]. 

Other herbs have also shown to be effective for the prevention of NDDs, such as Verbascum phlomoides and Solidago virgaureae [71]; however, there are limited studies available and evidence is scant for a conclusion about their effectiveness, mechanisms of action, and applicability in humans. One of the herbal compounds that we speculate to have neuroprotective effects is picein. In a previous study by our team, in silico studies on picein for the identification of putative biomolecular targets showed BACE-1 as the highest-ranked target enzyme for picein, which might indicate its neuroprotective role. An in vitro study on SH-SY5Y cells for the treatment of NB with picein showed that it recovered the mitochondrial activity to normal levels, which indicated the potential neuroprotective and antioxidative effect of this herbal agent [72]. In the next section, we review the medicinal properties of this compound and the available evidence regarding the antioxidative properties of this plant-derived natural biomolecule as well as its potential neuroprotective property. For reviewing the available evidence about picein, an online search was performed by the authors of PubMed, Google Scholar, Scopus, and Science Direct using the keyword “Picein”. All English articles have been studied by the authors separately and no date cutoffs were considered for the search. First, the titles of the articles were evaluated to indicate the articles related to the objectives of the present review. Studies on picein as a herbal compound were considered relevant. Where related to the subject of this review, their abstracts were studied. Then, the full texts of all English articles were evaluated. The articles found by different authors were compared and all related articles were included in this review. As the focus of this review was the antioxidative and neuroprotective effects of picein, we did not include the studies that focused on subjects other than the properties of picein, such as the methods of extraction, isolation, quantification, and preparation of this compound or its biological activity. 

## 4. Antioxidative and Neuroprotective Properties of Picein 

**Figure 1:**
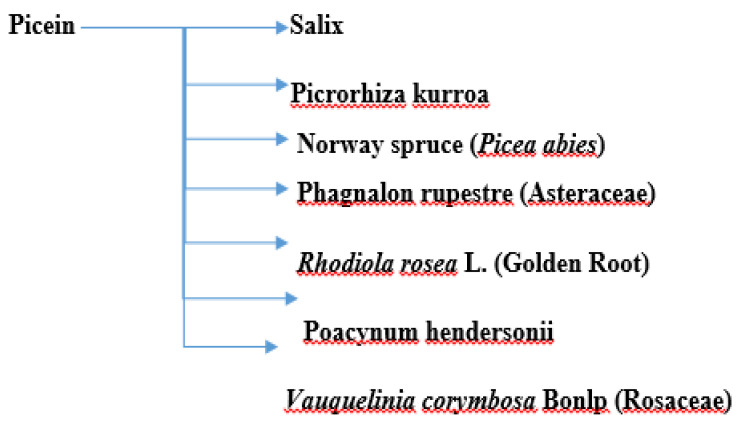
A summary of plants containing picein.

Picein (PubChem ID: 92123) is a phenolic (non-salicylic) glycoside extracted from different plant species with the formula C_14_H_18_O_7_ [73]. In our previous study, we showed the anti-inflammatory and neurodegenerative properties of NB [72]. However, the literature is scant and not centered in this regard. Below, we present the herbs from which picein can be extracted and evaluate their possible antioxidative, anti-inflammatory, and neuroprotective properties. Figure 1 shows a summary of the plants from which picein is extracted.

### 4.1. Willow (Salix)

One of the earliest records of treating inflammation and pain with herbal plants refers to the extracts of willow, Salix Sp., which belongs to the *Salicaceae* family. 

Analgesic, anti-inflammatory, antioxidative, anticancer, antidiabetic, antimicrobial, anti-obesity, antimigraine, cytotoxic, hepatoprotective, and neuroprotective activities have been documented for the Salix genus [74,75,76]. Salix is an aspirin precursor for which salicylic acid is extracted from water that is extracted from willow bark and leaves. It has also been used by animals and humans for its analgesic, antipyretic, and anti-inflammatory properties [77,78]. The antioxidative activity of Salix Sp. has also been identified and is mainly attributed to salicin [79], the mechanism of which is said to be the downregulation of the inflammatory mediators, tumor necrosis factor-α, and nuclear factor-kappa B [80]. Besides salicin, flavonoid and other phenolic compounds of Salix have also been identified to have anti-inflammatory properties and synergistic effects with coffee against scavenging free radicals and the inhibition of lipid peroxidation [81,82]. 

It is worth mentioning that Salix comprises several species such as *S. purpurea, S. daphnoides* clone 1095, *S. alba* clone 1100, *S. triandra, S. viminalis*, and *S. herbacea*. Each of these contains different concentrations of salicin, different compositions of phenols, and other compounds, for instance, acetylsalicortin was only found in *S*. *alba* [83]. Furthermore, an evaluation of 91 genotypes of the common species *Salix purpurea* showed that all were rich in salicylic glycosides (salicin, salicortin and tremulacin, flavanones, maringenin, chalcone isosalipurposide, and catechin), whereas picein and populin were only identified in 10% of them [84]. Heiska et al. extracted 1.61–31.08 mg/g d.m. from willow bark (*Salix myrsinifolia*) [85]. However, the comparison of the *S*. *daphnoides* and *S*. *purpurea* extracts showed the possible presence of picein in the former preparation [86]. The extracted compounds and their concentrations may not only vary based on the Salix genotype but also on the analytical (such as spectroscopy or chromatography) and extraction methods, as well as the plant part used, the age of the Salix, and the seasonal changes in their concentrations [87,88,89,90]. 

**Figure 2:**
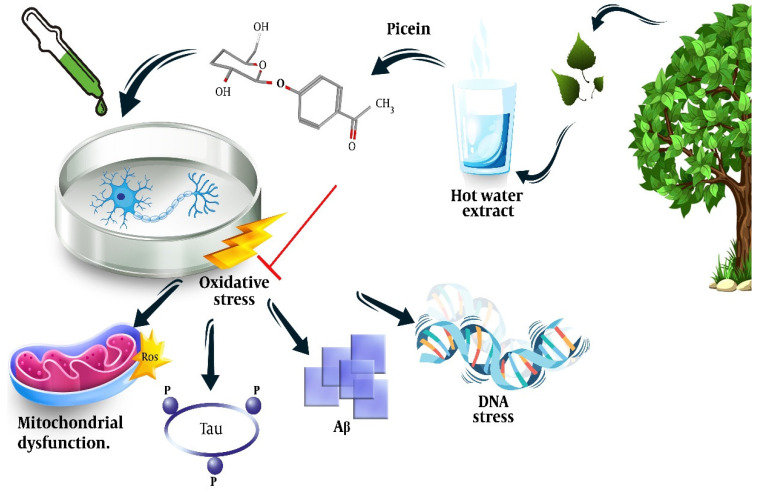
Isolation of picein from willow bark, its chemical formula, and illustration of the mechanisms behind its neuroprotective effects. Adapted from ref. [72].

Dou et al. extracted picein from a hot water extract (HWE) of willow bark by dissolving the freeze-dried HWE (5.2 mg) in pyridine (10 mL). Then, 0.7 mL of this solution and 0.1 mL xylitol (0.1 mg/mL) were treated with 0.2 mL BSTFA at 50 °C for 40 minutes, which resulted in a relative content ratio of 7.60 and a response factor of 3.04 [91]. The neuroprotective effect of Salix has been shown in previous studies [74]; however, we could not find any study other than one by members of our team that directly addressed the neuroprotective effect of the picein compound of Salix [72]. In this study, we showed that treatment of NB SH-SY5Y cells with picein decreased the menadione-induced ROS levels and recovered the mitochondrial activity to normal, indicating the potential of picein to be used as a neuroprotective agent (Figure 2). More details on this will be discussed in Section 5.

Although picein can be easily separated according to hydrophobicity differences and quantified using high-performance liquid chromatography [46], a few studies have separated the antioxidative effects of Salix Sp. based on the extracted compounds but failed to indicate any favorable effect for the picein compound. In a study by Jeon and colleagues, eight compounds were extracted from Salix hulteni and their cytotoxic effects were tested against brine shrimp and a human lung cancer cell line (H1299). The results showed that 4-Hydroxyacetophenone, picein, and sachaliside did not have cytotoxic effects [92]. In this study, Jeon and colleagues extracted 1.2 kg *S. huletni* with MeOH; suspended 220 g MeOH in water, partitioned with n-hexane, CHCL_3_, and n-BuOH to leave a residual water-soluble compound; evaporated each fraction; and tested the cytotoxic ability of each fraction [92]. Using n-butanol as a solvent resulted in the separation of picein with 93.6% purity [93]. Another study performed on immortalized human non-tumorigenic keratinocytes (HaCaT) also showed that the other compounds of Salix reticulata significantly inhibited cell proliferation, whereas such an effect was not observed for picein [94]. They also used the MeOH extract from aerial parts and showed that MeOH extract with a concentration of 100 µg/ml reduced cell viability by about 60%, but the separation of the flavonoids and compounds failed to show any effect on cell proliferation for picein [94]. Other compounds of Salix pseudo-lasiogyne twigs not including picein were also identified to have inhibitory effects against lipopolysaccharide-induced NO production in vitro (on microglial cells) and also significant antioxidative effects on lutathione reductase and superoxide dismutase production in vivo (on scopolamine-induced mice) [95]. Therefore, the available literature seems to show no antioxidative effect for the picein compound of the Salix genus; however, as described above, there are several factors that influence the presence and concentration of the compounds in this herb and the few studies in this regard have not reported the concentrations and extraction methods of picein in order to justify whether this concentration was sufficient for observing the antioxidative effect of this compound. Furthermore, the potential neuroprotective property of this compound is also missing from the literature, except for the one study performed by our team [72]. Therefore, more studies are required in this regard to be able to draw a conclusion about the properties of picein extracted from Salix. 

The review of the literature showed that picein is not only extracted from Salix but is also mentioned as a phenol glucoside compound extracted from other plants. Below, we describe the properties of picein extracted from other plants.

### 4.2. Picrorhiza kurroa

*Picrorhiza kurroa*, also known as Kutki, belongs to the Scrophulariaceae family and is a well-known herb in Ayurvedic medicine that is used for asthma, jaundice, fever, malaria, snake bite, and liver disorders, and is suggested to have antimicrobial, antioxidant, antibacterial, antimutagenic, cardioprotective, hepatoprotective, antimalarial, antidiabetic, anti-inflammatory, anticancer, anti-ulcer and nephroprotective properties [96,97]. A study by Morikawa et al. showed that picein extracted from the rhizomes of *Picrorhiza kurroa* had favorable results on the promotion of collagen synthesis (at 10–30 μM) without cytotoxicity observed at effective concentrations [98]. Studying the antioxidative activity of picein extracted as one of the two phenol glycosides of this herb identified no such effect for picein, whereas the other compound of these leaves, luteolin-5-O-glucopyranoside, showed significant antioxidative properties [99]. We believe that evidence is scant about the effects of picein extracted from this plant and further studies are required in this regard. 

### 4.3. Norway Spruce (Picea abies)

Picein and its aglucone piceol (4-hydroxy acetophenone) are phenolic compounds found in different parts of Norway spruces (*Picea abies*) [100]. In Norway spruce, picein contained 0.09–0.2% of the dry weight of non-mycorrhizal short roots [101] and 1.8–2.2% of dry weight in the spruce needles (which contained a 0.4–1.1% piceol concentration) [102,103]. Picein is considered the precursor of piceol and the effects observed from this phenolic compound are mainly through piceol release. Picein has also been suggested as an indicator of plant stress [104,105]. Ganthaler and colleagues evaluated the concentrations of different compounds in Norway spruce forest trees and showed that picein and stilbene concentrations were associated with the tree’s defensive power against fungal pathogens [106] and budworm [107]. Bahneweg and colleagues also showed increased picein in response to the fungal provenance of Norway spruce by Sirococcus conigenus [108]. The antimicrobial activity of picein against both Gram-positive and Gram-negative bacteria (*Staphylococcus aureus*, *S*. *epidermidis*, *S*. *typhimurium*, *Escherichia coli*, *Bacillus cereus*, *Klebsiella pneumoniae*, *Enterococcus faecalis*, and *Pseudomonas aeruginosa*) has also been detected, with minimum inhibitory concentration values of 16–64 mg/L [109]. 

Another similar tree is White spruce (*Picea glauca*), which also contains (the acetophenone glucoside) picein (the second most abundant metabolite with averages exceeding 10% of the foliar dry weight) that exerts favorable results against spruce budworm (*Choristoneura fumiferana*), a highly damaging forest insect pest [110]. Although the above-mentioned studies have confirmed the favorable effects of picein and piceol on the protection of Norway and White spruces against tree harm, the picein extracted from these plants has not been examined in cellular or animal studies to indicate the efficacy of this compound on other cells. 

### 4.4. Other Plants 

In addition to spruce, picein has been mentioned as a phenolic compound of several other plants, for instance, the aerial parts of Phagnalon rupestre (Asteraceae) [111], Ebenus pinnata [112], and *Rhodiola rosea* L. (Golden Root) [113,114]. However, the properties of this compound have not been investigated. 

Our literature search also found a few cellular studies on the random effects of plant-derived picein. Morikawa and colleagues showed that picein and several other compounds extracted from the flowers of Poacynum hendersonii could moderately promote adipogenesis of 3T3-L1 cells (a cell line derived from mice) [115]. Picein extracted from the aerial parts of Vauquelinia corymbosa Bonlp (Rosaceae) has shown favorable enzymatic activity against yeast and rat small intestinal α-glucosidases, which shows this compound is a potential source of the α-glucosidase inhibitor and is suitable for the development of new antidiabetic drugs [116]. Lai and colleagues have also shown that the guinea pig liver cytosolic beta-glucosidase can hydrolyze the plant glucoside, L-Picein, and its product, piceol [117], which suggests an ability to use this compound as an oral agent for this animal. However, further studies are required in this regard.

## 5. The Potential Role of Picein in the Treatment of Alzheimer’s Disease

As described in Section 2, Aβ42 peptide and NFT, composed of hyperphosphorylated Tau protein, are the two major neuropathological hallmarks of AD. Aβ aggregation is regarded as the precursor for the neurotoxicity of AD, which leads to increased assembly of the toxic and plaque-promoting Aβ42 peptide [118]. Enzymes, including BACE1 (a transmembrane aspartic acid protease) and γ-secretase, are involved in the generation of the Aβ peptide by APP cleavage [119,120] and their activity is directly associated with Aβ formation. Accordingly, pharmaceutical companies have performed and continue to perform clinical trials on the potential of the BACE1 inhibitors (MK8931, AZD-3293, JNJ-54861911, E2609, and CNP520) for the treatment of AD and downregulation of Aβ levels [120,121,122]. However, the initially promising BACE1 inhibitors failed in later stages of clinical trials because of the side effects, insufficient potency, or poor pharmacokinetics [123,124]. Further studies on novel classes of BACE1 inhibitors are being performed to investigate their potential as novel candidates for AD treatment [125].

In a previously mentioned study [72], we investigated and reported on in silico and in vitro studies of picein. The results of the in silico studies suggested BACE1 as a possible molecular target for picein. In addition, it was found to be structurally very similar to another molecule (gastrodin) that has shown to play a role in the reduction of memory deficit and neuropathology in a mouse model of AD [126]. Additionally, our in vitro studies demonstrated the potent neuroprotectant role of picein by neutralizing ROS generated by a neurotoxin [72]. These encouraging preliminary results strongly indicate that picein may have well-desired dual features, offering both a potential BACE1 inhibitory function and a potent neuroprotective effect. 

**Figure 3:**
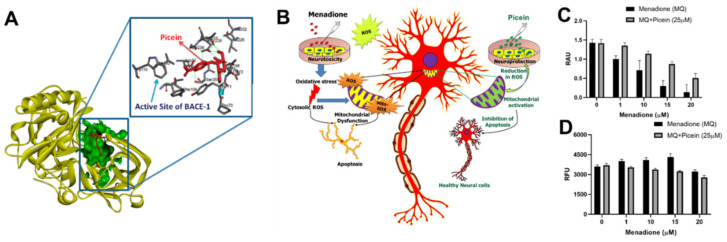
(**A**) In silico identification of putative biomolecular targets for the willow compound picein. BACE1 was found as the most probable target of picein. The docked pose of picein (red sticks) at the active site (green surface presentation) of BACE1 (yellow cartoon). Inset, the active site residues are shown as grey sticks. (**B**) An overview of the neuroprotective effects of picein: (**C**) increase in the level of cell viability, and (**D**) decrease in the level of ROS [72].

In the in silico studies, we used a pharmacophore mapping approach to predict the putative biomolecular targets of picein in silico with the PharmMapper web server [127]. BACE1 was predicted as the major molecular target of picein and molecular docking of picein to the BACE1 active site was successfully performed with AutoDock 4 [128]. The predicted binding affinity and Ki value (dissociation constant) of picein at the BACE1 active site were −5.94 kcal/mol and 44.03 µM, respectively, suggesting a modest affinity to the target. Key interactions of the docked pose of picein with the active site residues of BACE1 are shown in Figure 3A. These included seven hydrogen bond interactions as well as several hydrophobic contacts with the surrounding residues. 

This study also revealed that picein had strong neuroprotection properties. Here, picein was applied to evaluate its therapeutic impact on SH-SY5Y NB cells (Figure 3B–D). The observed results demonstrated the role of picein in neutralizing the damage caused by ROS (neurotoxin) and thus greatly influencing the survival of NB cells. Additionally, the CNS is predominantly susceptible to oxidative stress as it utilizes oxygen (O_2_) at a higher rate with low concentrations of antioxidants and related enzymes, although they have a high content of oxidation-susceptible biomacromolecules and polyunsaturated lipids. Consequently, a strong neuroprotection molecule with neutralizing effects on oxidative stress is well-desired for neuronal cell survival.

## 6. Conclusions

One of the most prominent medical concerns in the world today is related to NDDs for which no definite treatment has yet been suggested. With an aging population and an increase in environmental risk factors due to industry, incidences of NDDs are on the rise. Therefore, research has focused on alternatives to pharmacological medicine that can only relieve patients’ symptoms rather than on finding a method that can prevent the occurrence of NDDs and slow down disease progression. Considering the role of inflammation, oxidative stress, and neurodegeneration in the pathogenesis of NDDs, the therapeutic and preventive roles of several antioxidative agents, including nutritional and herbal medicines, have been investigated in cellular and molecular studies. 

Picein is one of the active compounds of several plants and herbs with a potential for neuroprotection. Salix, an aspirin precursor, has shown significant anti-inflammatory, antioxidant, and neuroprotective properties, and picein is one of the phenolic compounds that can be extracted from the water extract of willow bark. In a previous study, our team has shown that treatment of NB SH-SY5Y cells with picein decreased the menadione-induced ROS levels and recovered mitochondrial activity to normal, indicating the potential of picein as a neuroprotective agent. However, there are no other studies on the direct neuroprotective effect of picein. A few studies have reported the antioxidative properties of Salix Sp. based on the extracted compounds in cellular models but failed to indicate any favorable effect for the picein compound. *Picrorhiza kurroa* is another herb from which picein is extracted. However, the one study that evaluated the effect of the extracted picein from this herb failed to show anti-inflammatory and antioxidative properties for this compound. These studies do not provide sufficient evidence for a definite conclusion, either for the anti-inflammatory and antioxidative properties of picein or its neuroprotective potential. Picein has also shown favorable antifungal and antibacterial properties in spruce trees. Although these plant studies can be appropriate evidence for further studies, no studies have evaluated the effects of the picein extracted from these plants on other cells; therefore, we cannot draw definite conclusions in this regard. As described in this review, most studies have proven that picein has no cytotoxic properties, which confirms its safety; however, the exact mechanism of the action and efficacy of picein on NDDs should be investigated in depth in the future. 

## 7. Future Directions

The treatment of NDDs is still a major concern and therapeutic agents that tackle the pathophysiology of the disease are required for effective treatment. In the present review, we presented evidence from a few studies that confirm the antioxidative and neuroprotective role of picein in addition to its lack of cytotoxicity. Therefore, this plant-derived natural biomolecule may be a safe and effective agent for the treatment of NDDs. Nonetheless, the number of studies is limited in this regard and more studies are required on animals to prove these properties and propose this agent as a novel therapeutic agent. We also recognize the delivery challenges of transiting drug molecules across the blood–brain barrier in the treatment of NDDs and methods addressing this issue are being developed in parallel to the discovery of new, promising compounds for the same. 

## Data Availability

Not applicable.
